# Pseudotyping Lentiviral Vectors: When the Clothes Make the Virus

**DOI:** 10.3390/v12111311

**Published:** 2020-11-16

**Authors:** Alexis Duvergé, Matteo Negroni

**Affiliations:** 1 Université de Strasbourg, CNRS, Architecture et Réactivité de l’ARN, UPR9002, 67000 Strasbourg, France; duverge@unistra.fr; 2 Interdisciplinary Thematic Institute (ITI) InnoVec, Université de Strasbourg, 67000 Strasbourg, France

**Keywords:** pseudotyping, lentiviral vectors, envelope proteins, gene therapy

## Abstract

Delivering transgenes to human cells through transduction with viral vectors constitutes one of the most encouraging approaches in gene therapy. Lentivirus-derived vectors are among the most promising vectors for these approaches. When the genetic modification of the cell must be performed in vivo, efficient specific transduction of the cell targets of the therapy in the absence of off-targeting constitutes the Holy Grail of gene therapy. For viral therapy, this is largely determined by the characteristics of the surface proteins carried by the vector. In this regard, an important property of lentiviral vectors is the possibility of being pseudotyped by envelopes of other viruses, widening the panel of proteins with which they can be armed. Here, we discuss how this is achieved at the molecular level and what the properties and the potentialities of the different envelope proteins that can be used for pseudotyping these vectors are.

## 1. Gene Therapy Using Viral Vectors

According to the definition provided by the NIH Genetics Home Reference, gene therapy is an experimental technique aimed at treating or preventing a disease by using genes [1]. This can be achieved by various means. When the disease is of genetic origin and, particularly, when it is caused by a single defective gene, the ultimate goal is replacing the defective gene with a wild-type one. This has been possible only recently with the development of powerful genome editing techniques [2,3,4]. Although, these are not applicable routinely and alternative approaches are followed, the most common of which is the introduction of a gene conferring a dominant wild-type phenotype to the modified cell [5]. Whatever the approach followed, gene therapy relies on the use of vectors that allow the efficient genetic modification of cells, or tissues, combined with a high specificity for the target cells to reduce adverse effects [6]. Introducing exogenous genetic material in cells is efficiently performed by cellular “parasites”—phages for bacteria or viruses for eukaryotic cells. In particular, the vast range of human viruses provides a large panel of promising tools for vectorization (by transduction) in sight of intervention on human cells. How to reprogram human viruses for the purposes mentioned above is a major challenge in molecular medicine.

A main watershed in gene therapy is whether the genetic modification of the cell must be carried out ex vivo or in vivo. If the cells’ target for the therapy can be isolated from the patient, modified ex vivo, and reinfused in the patient, essentially no specific tropism is required for the vector since the cells to modify are the only ones it comes into contact with [7,8,9,10]. In this case, the vectors can therefore carry pan-tropic envelope proteins such as, for example, the vescicular stomatitis virus (VSV) envelope protein G (see below). If, in contrast, the modification of the cells must be carried out in vivo, a high specificity for the target cells is required to avoid off-target transduction. The nature of the envelope proteins carried by the viral vector is the major determinant for the specificity of transduction.

Most gene therapy clinical trials carried out to date have relied on the use of adeno-associated vectors (AAVs) or retroviral vectors, which might be derived from γ-retroviruses or lentiviruses [11]. Modification of cells in vivo (liver, muscles, central nervous system and retina) has been restricted to the use of AAV-derived vectors, while ex vivo approaches (for the genetic modification of T cells and of human hematopoietic stem and progenitor cells) have relied on the use of vectors derived from murine γ-retroviruses and human lentiviruses. The neat division between clinical trials where AAV vectors have been used and those involving retroviral vectors is in part explained by the natural tropism of the viruses from which these vectors have been constructed.

AAV are non-enveloped non-integrative single-stranded DNA viruses of the *Parvoviridae* family. They require coinfection by adenoviruses to replicate and are non-pathogenic for humans. They infect replicating as well as quiescent cells and enter into the target cells by interaction with sialic acid, heparan sulfate, or galactose present on their surface, and therefore possess a large tropism. Differences in the capsid protein of AAV determine cell type-specific preferences and define the existence of the eleven serotypes of this virus. For gene therapy, according to the type of target tissue, serotypes that naturally target that type of tissue, when such serotypes exist, are the preferred choice for building a viral vector. To date, in gene therapy, eight serotypes (1–2 and 4–9) have been used to orient viral transduction toward the tissue of interest [12].

In sharp contrast to AAV, γ-retroviruses and lentiviruses do not present different serotypes and no variation in tissue specificity is found for these viruses, which both target blood cells. For example, in human immunodeficiency virus (HIV), despite its impressive genetic diversity, which is particularly high at the level of its envelope proteins, infection remains essentially restricted either to CD4+/CCR5+ or CD4+/CXCR4+ cells. However, an interest of retroviral-derived vectors (and therefore of lentiviral-derived vectors as well) comes from the possibility of replacing the original envelope proteins with those of other viruses, a process called pseudotyping. In this review article, we focus on the perspectives on which pseudotyping lentiviral-derived vectors (LV vectors) open and how this is achieved.

## 2. Lentiviruses and Gene Therapy

Retroviruses are enveloped viruses that integrate in the infected cell. This property has made of these viruses the preferred choice for developing vectors when the expression of the transgene must be stable or when the transgene must be inherited by the progeny of the transduced cell. For these reasons, retroviral vectors have been chosen for the expression of transgenes in hematopoietic stem and progenitor cells (HSPCs) and, more recently, they have been used for the transduction of peripheral blood cells for the generation of CAR-T cells [13]. Gammaretroviral vectors derived from Moloney murine leukemia were used for the earliest gene therapy assays using retroviral vectors. They have been successful in the treatment of several primary immunodeficiencies, such as the X-linked severe combined immunodeficiency (SCID) or the adenosine deaminase deficiency-induced SCID [14,15,16], and they have been employed in the treatment of the Wiskott–Aldrich syndrome and of X-linked chronic granulomatous disease [17,18,19]. However, γ-retroviral vectors have been progressively replaced by the lentiviral vectors (LV vectors), mostly due to the lower levels of induction of the innate immune response they trigger [20,21] and, in particular, for biosafety reasons. Indeed, LV vectors predominantly integrate in transcription units [22], rather than in regions controlling gene expression as promoters and enhancers that are, instead, the preferential sites of integration for gammaretroviral vectors [23,24]. This difference has been shown to lead to a lower probability for lentiviruses to cause insertional oncogenesis [25,26]. LV vectors have thus been used in most recent trials, always for the treatment of blood diseases. Besides treating the same diseases with these new vectors as are treated with γ-retroviral vectors mentioned above [27,28,29,30,31], β-thalassemia [32], Fanconi anemia [33], metachromatic leukodystrophy [34,35], mucopolysaccharidosis type I [36], adrenoleukodystrophy [37] and sickle cell disease [38] have also been made the object of clinical trials using LV vectors.

## 3. Molecular Biology of Lentiviruses

Lentiviruses belong to the subfamily *Lentivirinae* of retroviruses [39]. They are considered as “complex” retroviruses, due to the presence of additional genes, compared to other retroviruses. As all retroviruses, they are enveloped integrative viruses. The viral particle is constituted by a spheric matrix shell that lies immediately underneath the lipid bilayer, which consists of a patch of the cell membrane that is carried over during viral budding from the infected cell [40]. More internally, a fullerene-shaped core [41] contains the genomic RNA that is constituted by a single-stranded positive-sense molecule, present in two copies in the viral particle, in a dimeric form. Upon infection of the host cell (that occurs after recognition of a specific receptor on the surface of the cell) the viral capsid enters the cytoplasm. The availability of the nucleotides, to which the capsid is permeable, allows the initiation of reverse transcription. This results in the conversion of the genomic RNA into double-stranded DNA which is then integrated in the cell genome [42,43,44,45].

Where and when this conversion occurs and is achieved remains a matter of debate. The traditional view according to which reverse transcription was completed in the cytoplasm or at the nuclear pore, followed by the dismantling of the capsid core and the import of the preintegration nucleoprotein complex [46,47,48,49], has recently been challenged by the observation of intact or almost-intact cores, as well as the detection of ongoing reverse transcription in the nucleus [50]. However, irrespective of the form under which the genetic material is imported into the nucleus, the import occurs in an active manner, through the interaction of the viral capsid protein p24 with the cellular protein cyclophillin A and the cellular splicing factor CSPF6 [51,52,53]. This interaction leads to the use of the nuclear import pathway relying on the pair of nuclear pore proteins Nup153/Nup358 and transportin 3 (TNPO3) [54]. This complex system allows lentiviruses (in the specific case detailed above, human immunodeficiency virus type 1 (HIV-1)) to infect non-replicating cells. This not only allows LV vectors to deliver transgenes to cells that naturally do not replicate, but also can be exploited for transducing cells, such as HSPCs, that must be kept in a quiescent state to avoid their differentiation and loss of pluripotency. Retroviruses as γ-retroviruses are instead unable to enter the nucleus of the infected cell and require the disassembly of the nuclear membrane at mitosis for reaching the genome of the infected cell for integration.

Integration is carried out by the viral enzyme integrase with poor sequence specificity for the selection of the integration sites, although preferential types of genomic regions (as, for example, regions where actively transcribed genes are located, or the proximity with respect to transcription start sites) can be defined for the different types of retroviruses [55,56]. The reverse transcription product, integrated in the genomic RNA of the infected cell, is called a provirus. The provirus is flanked by the terminal repeated regions (LTRs) that contain the viral promoter sequence (see below). Transcription from the LTR in 5’ will lead to the synthesis of the new genomic RNA as well as the viral proteins required for infection to be continued. At the moment of assembly of the viral particle, the dimers of viral genomic RNA will be packaged in the budding particle [57]. The particle will also incorporate the envelope proteins at their surface, as detailed below, and be released in the extracellular space as an immature particle. Activation of the viral protease in the immature particle, will then lead to viral maturation and the production of an infectious virus [40].

## 4. Molecular Bases for the Making of LV Vectors

### 4.1. Structure of the Genomic RNA

LV vectors are generally derived from the best characterized lentivirus—human immunodeficiency virus type 1 (HIV-1). Lentiviral infection, detailed above, is conceptually composed of two phases. Entry and the conversion of the genomic RNA (gRNA) into DNA that will be integrated in the cell’s chromosomes are considered as the “early phase” of the infectious cycle. With the exception of entry, which depends on the nature of the envelope employed in the viral vector, all the steps of the early phase of HIV-1 infection are carried out essentially in the same manner during LV vector-mediated transduction. The late phase, constituted by the production of the gRNA and of the viral proteins, is instead absent in the case of LV vector transduction.

**Figure 1:**
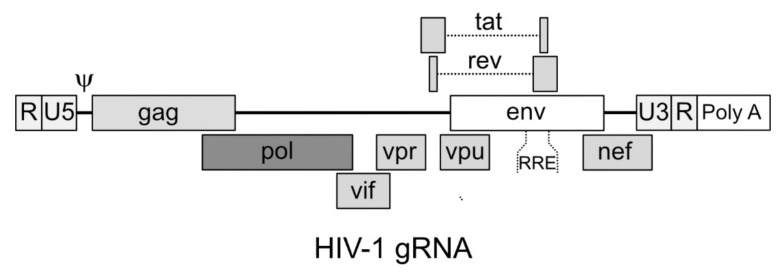
Organization of the human immunodeficiency virus type 1 (HIV-1) genomic RNA. U3, unique sequence 3’; R, repeated sequence; U5, unique sequence 5’; Ψ, indicates the packaging and dimerization sequences; RRE, Rev responsive element. The PPT sequences as well as the primer binding sequence (PBS) region are not shown.

The viral gRNA of HIV-1 is characterized, proceeding from 5’ to 3’, by the terminal repeated sequence R, the unique sequence in 5’ U5; then, contiguous to U5, are found the 18 nucleotides that constitute the sequence to which the tRNA Lys^3^ anneals for priming reverse transcription (primer binding sequence (PBS)) [58] followed by an untranslated 5’ region that is responsible for the dimerization of the gRNA and its packaging in the viral particle [57]. Then, the main three genes (*gag*, *pol* and *env*) follow, overlapping the sequences for the auxiliary proteins Vif, Vpr and Vpu, as well as the proteins Tat and Rev (Figure 1). Finally, partially overlapping with the 3’ portion of *env*, the Nef coding sequence is found, followed by the unique sequence in 3’ U3, the repeated sequence R and the polyA tail [59]. The sequences required for priming the synthesis of the second strand of DNA (3’ and central polypurine tracts, -3’ PPT and cPPT, respectively) are located immediately upstream of the U3 sequence and in the 3’ end portion of *pol*, respectively [60,61]. The Rev Responsive Element (RRE) sequence that, when bound by the Rev protein allows the export of partially unspliced RNAs from the nucleus, is located in the portion of *env* encoding the gp41 protein [62].

**Figure 2:**
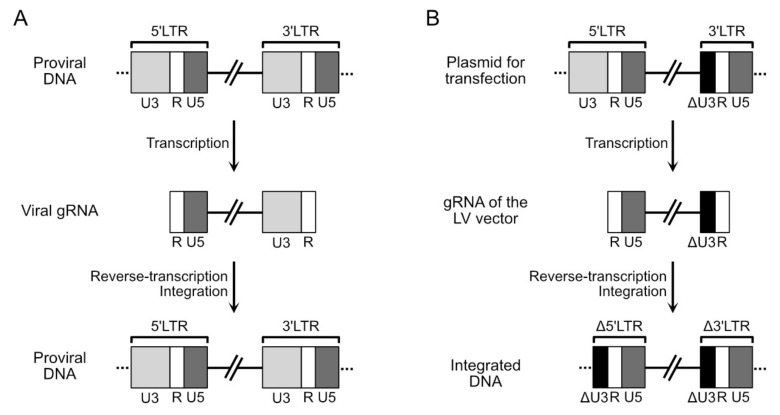
Schematic representation of the structure of the genomic forms of the viral DNA during natural infection (panel (**A**)) or during transfection and transduction by a self-inactivating (SIN) lentiviral-derived (LV) vector (panel (**B**)).

To generate the gRNA of the LV vector, the viral gRNA is modified by removing all the coding sequences for the viral proteins and leaving the elements required in *cis* for genomic RNA packaging, reverse transcription and integration. Specifically, the gRNA of the vector must contain: the PBS sequence; the 3’ PPT and cPPT sequences; the region (located in the 5’ untranslated portion of the genome) responsible for the packaging and dimerization of the genomic RNA; the RRE sequence; the repeated terminal sequence R and the sequence U5, which are required for achieving reverse transcription and integration [63]. The U3 sequence, instead, is only partially preserved, since a large deletion (approximately half of its total length) is made in this sequence [64]. The deletion is essential for inactivating, in LV vectors, the promoter activity of U3, generating what are known as self-inactivating (SIN) vectors [64]. In natural infections, the U3 sequence is located inside the LTR sequences, present at both ends of the proviral DNA (Figure 2A). The U3 sequence located in the 5’ LTR contains the promoter that is used to drive the transcription of the genomic RNA. The genomic RNA contains only the U3 sequence of the 3’ LTR (Figure 2A). After reverse transcription of this genomic RNA, the LTRs are again generated (Figure 2A). In the case of SIN LV vectors, the U3 sequence of the 3’ LTR carries the deletion (in black in Figure 2B) and it will be this sequence that will be present in the genomic RNA. After reverse transcription, this deleted version of U3 will be present in both LTR, the 5’ and the 3’ regions (Figure 2B). Transcription is thereby no longer possible from this proviral DNA, since the promoter in the U3 sequence in the 5’ LTR is not functional. Taking into account these requirements, the gRNA of the LV vector can accommodate up to 8 kb of exogenous sequences.

**Figure 3:**
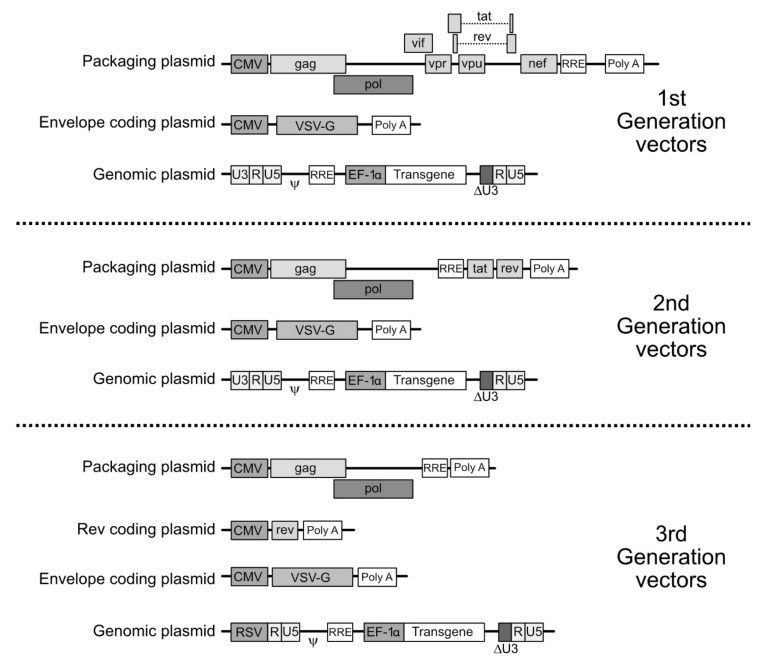
The various generations of lentiviral vectors. Top panel. Plasmids employed for constructing first generation lentiviral vectors. Three plasmids are employed. (1) A packaging (or transcomplementation) plasmid encodes the Gag, Pol, Vif, Tat, Rev, Nef, Vpr proteins under the control of the Cytomegalovirus (CMV) promoter. (2) The envelope protein(s) (the vescicular stomatitis virus (VSV) G protein in the example given) is encoded by an expression plasmid under the control of the CMV promoter. (3) Finally, the plasmid leading to the synthesis of the genomic RNA (“genomic plasmid”) contains the sequences required in cis for the packaging and reverse transcription of the RNA. It also contains the sequence of the transgene under the control of an internal promoter (EF1α in the example). The expression of the genomic RNA is driven by the 5’ terminal repeated regions (LTR). The sequence U3 in the 3’ LTR is partially deleted, inactivating the promoter present in U3 generating self-inactivating (SIN) vectors. Middle panel: 2nd generation vectors are built by triple transfection of the producer cells. In this generation of LV vectors, the packaging plasmid only encodes Gag, Pol, Tat and Rev, increasing the level of biosafety (the auxiliary proteins Vif, Nef and Vpr are absent). Bottom panel, third generation of vectors. The packaging plasmid is split in two plasmids, one encoding (under the control of the CMV promoter) the Gag and Pol sequences and carrying the Rev responsive element (RRE), the second encoding the Rev protein (also under the control of the CMV, in the example given). In the genomic plasmid the 5’ LTR sequence is replaced by the sequence of a chimeric LTR where the U3 sequence is replaced by that of a heterologous promoter (the Rous Sarcoma Virus-RSV-promoter in the example given). Finally, "next generation" vectors have also been elaborated, but since they differ considerably from one another, no "synthetic" drawing summarizing them is provided. The main improvement consists of the splitting of the coding sequences in a larger number of plasmids, for increasing biosafety.

For the generation of the LV vector particle, the plasmid leading to the synthesis of the gRNA is cotransfected with transcomplementation plasmids leading to the synthesis of the viral proteins. Depending on which generation of LV vectors is considered, the structure of the plasmids varies as well as which viral proteins are provided (Figure 3). In this setting, in order to change the tropism of the viral vector through pseudotyping, the plasmid encoding the envelope proteins will be chosen to carry the desired, non-HIV, envelope protein coding sequences.

### 4.2. Mechanism of Entry in HIV-1

The need for pseudotyping LV vector particles comes, as mentioned above, from the difficulty of modifying the mechanism of viral entry of the natural HIV envelope proteins. HIV-1 encodes two envelope proteins—gp41 and the gp120. The gp41 is a transmembrane protein that, associating in a non-covalent manner to the gp120 (that is located on the external side of the virus), forms an unstable heterodimer [65]. Three of these heterodimers associate to form a trimer of dimers that constitute the viral spike [65]. Viral entry occurs by fusion of the cell and viral membranes, carried out by the viral envelope proteins. For this, the spike interacts, through the gp120 component, with the natural HIV-1 receptor, the CD4 molecule [66,67]. This triggers a conformational change that leads to the generation in the gp120 of a binding site for the HIV coreceptor, generally the transmembrane protein CCR5, or CXCR4 [68,69,70,71,72]. This second interaction is responsible for another structural rearrangement of the gp120/gp41 dimer that releases the gp41 from the interaction with the gp120 [73]. The gp41 that was maintained in a metastable state by the interaction with the gp120 inserts its highly hydrophobic N-terminal portion, called “fusion peptide”, in the internal portion of the spike, in the membrane of the target cell [74]. Once this has occurred, the gp41 folds back on itself to reach the most stable conformation possible. This brings the fusion peptide (still inserted in the cell membrane) in proximity of the viral membrane leading to the fusion of the membranes [75,76,77,78] and to the creation of a pore that, once enlarged, allows the entry of the viral core into the cytoplasm. Because of the complex series of conformational transitions required for the functionality of the envelope, the interactions between the two Env proteins must be based on highly unstable equilibria that are extremely difficult to retain if one wishes to modify this system, in order to redesign the tropism of the virus. Therefore, changing the tropism of a HIV-derived LV vectors is a fairly difficult goal to achieve through the modification of the natural HIV envelope proteins. However, the relative ease with which a LV vector can be efficiently pseudotyped by exogenous viral envelope proteins provides alternative solutions to bypass these difficulties.

### 4.3. Mechanism of Recruitment of HIV-1 Envelope Proteins on the Surface of the Virus: Bases for Pseudotyping

The HIV-1 particle is enveloped by the plasma membrane of the cell from which the virus has budded. Consequently, the lipid and protein compositions of the viral membrane reflect that of the infected cell at the site of budding. The peculiar composition of lipids and proteins of the viral particle with respect to that of the cell, suggests that viral budding occurs in specific regions of the membrane with a particular lipid composition. To be incorporated in the budding viral particle a protein must be addressed to the cell compartment where viral budding occurs [79]. These observations set the bases to conceive the possibility of pseudotyping lentiviral particles.

HIV-1 assembles at lipid rafts, areas of the membrane enriched in cholesterol and sphingolipids. This lipid composition tends to be enriched in glycosylphosphatidylinositol-anchored proteins at the site of budding [80]. Potentially, any protein with a high “affinity” for lipid rafts has a higher probability than the average protein to be found on the viral particle. This strategy allows the virus to reduce immunogenicity during natural infections and, consequently, also leads to the generation of poorly immunogenic HIV-derived LV vector particles. Assembling at lipid rafts indeed provides a lipid membrane of the viral particle enriched in proteins such as CD46, CD55 and CD59 [81,82,83,84] which are known to inhibit complement activation [85,86,87]. Accordingly, when the virus is produced in glycosylphosphatidylinositol-anchors deficient cells, it becomes sensitive to degradation by the immune system [88].

In HIV-1, the envelope proteins are recruited at lipid rafts through the interaction between the cytoplasmic tail of the gp41 and the precursor polyprotein Pr55 Gag, which is localized at the lipid rafts thanks to the myristoyl group that is present at its N-terminus [89]. It also has been shown that the acylation of the transmembrane domains of proteins was sufficient to address these proteins to the lipids raft [90,91]. Acylated proteins potentially prevail in viral particles that bud from lipid raft rich areas. This characteristic of acylated proteins provides a “tool” to induce pseudotyping in LV vectors. Accordingly, VSV glycoproteins are acylated [92], as well as the E2 envelope glycoprotein of alphaviruses such as Semliki forest virus and Sindbis Virus [93,94]. All these envelope proteins efficiently pseudotype LV vectors. For other viral envelope proteins, such as the rabies ones for instance, the molecular mechanism leading to pseudotyping LV vectors is known in much less detail, but it is logical to expect that, also in this case, pseudotyping occurs through addressing these proteins to lipid rafts.

## 5. Dressing LV Vectors (Pseudotyping)

**Figure 4:**
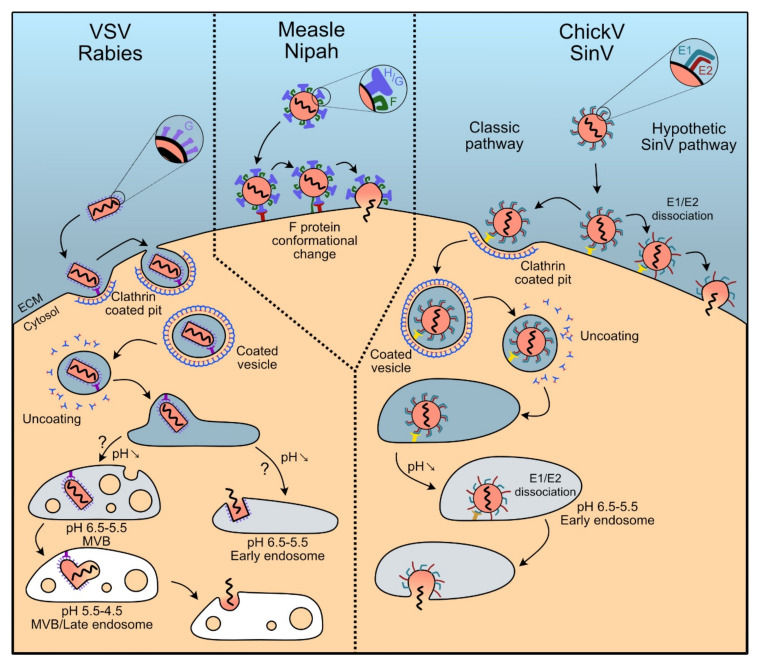
Outline of the mechanisms of viral entry by the main envelope proteins that can be used to pseudotype LV vectors. ECM: Extracellular Medium; MVB: Multivesicular Body.

Pseudotyping LV vectors with envelope proteins of different viruses allows combining the properties of lentiviruses with those of viral entry of other viruses. The envelope proteins of several types of viruses have been shown to be able to pseudotype LV vectors. Among these viruses, some possess a large tropism and therefore the use of their proteins for treatments in vivo cannot be envisaged. However, some of these proteins can be used as starting platforms for engineering variants that specifically target a desired cell population. Envelope proteins from other viruses, instead, present a tropism restricted to certain types of cells (neurons, for example), and can be employed “opportunistically” when these cell types constitute the target of the intervention strategy. Envelope proteins from several viruses have been described to successfully pseudotype LV vectors. Those for which the molecular mechanism has been elucidated in more detail fall into three viral families and are presented here (Figure 4 and Table 1).

### 5.1. Rhabdoviruses: Clathrin-Dependent Endocytosis

#### 5.1.1. Vescicular Stomatitis Virus

Pseudotyping LV vectors by the vescicular stomatitis virus envelope glycoprotein G is the most common approach for creating cell lines [117]. VSV is an enveloped virus from the *Rhabdoviridae* family. It expresses the G glycoprotein on the envelope surface. The first hypothesis to explain the large tropism of the virus suggests that it might use not only a specific, widespread, receptor but that it possibly also uses alternative receptors. The first receptor described for VSV-G were the phosphatidylserines, phospholipids that are a main components of plasma membranes [118,119] present at the surface of almost all cell types [119]. However, more recent work showed that treatment with annexin V, a specific ligand for phosphatidylserines, did not inhibit infection by VSV [120]. In addition, the same work showed an absence of correlation between the content of phosphatidylserines in the plasma membrane and the efficacy of infection by VSV [120]. In 2013 it was demonstrated that the main receptor promoting VSV entry are members of the LDL-Receptor (LDL-R) family [95]. These receptors are involved in the regulation of the homeostasis of cholesterol in mammalian cells and are ubiquitously expressed [121,122]. Once bound to the cell membrane, the VSV envelope protein VSV-G triggers clathrin-dependent endocytosis [123], typical of the *Rhabdoviridae* family, followed by pH-dependent fusion of endosomal and viral membranes [124,125], leading to the release of the capsid in the cell, although it is still debated whether membrane fusion occurs in the early endosome or in the late endosomes/multivesicular bodies [126,127]. To begin fusion, a conformational change of the G protein is required first to anchor the virus into the cell membrane and then to operate a physical connection between the two lipid bilayers that ultimately allows membrane fusion [128].

The quasi-universal tropism provided by the glycoproteins G of VSV makes it difficult to conceive a safe manner for the systemic inoculation of these vectors in patients, because of obvious problems related to off-target delivery. When the natural biodistribution that follows the systemic administration of vectors is favorable, as for example when the liver is the target of the therapy, LV vectors pseudotyped by the VSV envelope protein G have proved to be successful for in vivo treatment, as for the case of the induction of the expression of the coagulation factor IX (FIX) in mice and hemophilic dog models [129,130]. Alternatively, their local administration can be considered in vivo, as it has been shown for colorectal administration in mouse models [131]. However, to date, pseudotyping LV vectors by the VSV envelope protein G for gene therapy is employed, in the majority of the cases, for transduction ex vivo [132,133].

#### 5.1.2. Rabies Virus

Rabies viruses (RVs) are negatively stranded RNA Rhabdoviruses, with a natural tropism for neurons. Accordingly, the use of their envelope proteins for pseudotyping LV vectors is strictly related to intervention on these cells. RVs infect neurons through their terminal axons and spread through the synapses in a retrograde direction, a feature that is maintained when RV-pseudotyped LV vectors are used [134]. Recognition of the receptor is ensured by the G protein, which interacts with a panel of different receptors, all expressed on neurons. After receptor recognition, RV particles are endocytosed following a clathrin-dependent uptake [135]. The internalized vesicles then fuse with the early endosomes, as a consequence of the acidification of the endosome, with a VSV-like mechanism [123] (Figure 3).

The first receptor described for RV was the nicotinic acetylcholine receptor (nAChR) [97]. This receptor is present at a high density in neuromuscular junctions [136]. Another receptor used by RV is CD56 (or NCAM) [98], involved in the adhesion of neural cells, the development of neurites and the synapses’ plasticity. However, CD56 is also abundant on natural killer cells, raising a concern about the specificity of its use. RVs have also been shown to interact with the nerve growth factor receptor (NGFR) superfamily, the p75NTR (Low-affinity Nerve Growth Factor Receptor: LNGFR) [99]. However, despite this interaction, infection is limited to around 20% of neurons, while more than 80% are p75NTR positive [137]. These results tend to show that the LNGFR is not the most important receptor for RV uptake. Finally, mGluR2, abundant in the central nervous system [100], has also been described as a receptor for RV. Indeed, it has been observed that RV and mGluR2 are internalized into cells and transported to early and late endosomes in close association, suggesting their functional interaction.

In conclusion, the efficient transduction by the RV G glycoprotein involves a wide panel of receptors, but is strictly limited to neurons. This characteristic can be exploited to transduce specifically neurons. Indeed, it has been shown that an injection of RV-pseudotyped LV vectors directly in the muscle can lead to gene transfer in the spinal cord motoneurons while, under the same conditions, a vector pseudotyped with VSV-G have transduced muscle cells around the injection site, without any expression in the spinal cord neurons [134]. However, crossing the hemato-encephalic barrier by LV vectors in adults is mostly restricted to some more permissive areas as the median eminence (hypothalamus), pituitary, choroids plexus or pineal gland [138], limiting the use of RV-pseudotyped LV vectors.

### 5.2. Paramyxoviruses: Splitting Binding and Fusion in H and F Proteins

#### 5.2.1. Measles Virus

Measles virus (MV) is an enveloped, single-stranded RNA virus of negative polarity belonging to the family of *Paramixoviridae*. It is responsible for the measles disease in humans, against which extensive vaccinal strategies were developed over decades. Its envelope glycoproteins confer a wide cell tropism. Recognition of the receptors is ensured by the H protein (hemagglutinin) present at the surface of the virus. Once the virus is bound to the target cell, membrane fusion is ensured by the F protein (where F stands for fusion). For this, F undergoes a first conformational change that allows it to anchor the cell membrane and, subsequently, start the fusion [139], which is achieved by bringing the viral and cell membranes in close proximity [140]. As for all *Paramixoviridae*, the mechanism of membrane fusion is pH independent and it occurs directly at the plasma membrane [141] (Figure 3).

The first receptor to be identified for measles was CD46, a molecule present on the surface of most human cells except erythrocytes [142,143,144]. Consequently, the use of these envelope proteins would result in an extremely large tropism, conferring only a little advantage with respect to the use of VSV-G. Furthermore, it appears that CD46 is not the only receptor for measles virus, since passages in cell culture of the MV on lymphoblastoid cell lines led to the selection of a strain capable of infecting cells not expressing CD46 [101,102,103]. This laboratory strain had the ability to infect lymphocyte cell lines through the use of the receptor SLAM (also named CDw150) [104,105,106]. However, SLAM is also expressed on some subsets of B and T human cells. Therefore, the problem of the lack of specificity encountered with CD46 is still present, although considerably reduced, if pseudotyping is performed with an envelope issued from a SLAM-tropic strain. Finally, for the wild-type MV and for the strain used in vaccinal approaches (the Edmonston laboratory strain), another receptor can be used—nectin-4 [145,146]. Nectins are responsible for calcium-dependent cell adhesion and are therefore found on epithelial cells. Nectin-4 is found overexpressed on the surface of tumor cells in some ovarian [147] or lung [148] cancers and could therefore be used for treatment of cancers expressing this receptor. Therefore, three types of receptors have been identified for this virus. While for two of them the tropism is too large for their use, for the third one it is possible to envisage its use if the cells to target match the natural tropism of this viral variant.

The H protein can also be used as a basis for redirecting viral tropism. In this sense, mutations that abolished binding to CD46 and SLAM were identified, an important issue for reducing off-target delivery [149]. The first relevant case of complete MV retargeting consisted of the modification of the H glycoprotein, by inserting a single-chain antibody directed against CD38 or EGFR to be used as an oncolytic vector [150]. The engineered viruses mediated efficient infection through their respective receptors targeted, but not through, CD46 or SLAM. This work showed the possibility of a complete specificity change of the H protein. Single-chain antibodies are not the only way to retarget the specificity of the MV envelopes, though. It has been shown that the insertion of designed ankyrin repeat proteins (DARPins), consisting of at least three ankyrin repeats, in H protein can allow the retargeting of a MV-pseudotyped LV vectors [151]. In addition to an efficient retargeting, the H protein fused to a DARPin has a higher level of surface expression than the H proteins fused to a single-chain antibody [151].

The major obstacle to the development of a pseudotyping approach based on the use of MV envelope proteins is constituted by the neutralization of the vectors, due to the large vaccinal coverage against measles virus present in the human population. Since H protein seems to be the main target of the immune response [152], a promising approach is constituted by the introduction of mutations in this protein that allow escape, to a certain extent, from immune recognition while preserving its functional activity [153].

#### 5.2.2. Nipah Virus

Another Paramyxovirus, the Nipah virus (NiV), a virus that can cause severe flu-like disease in human with potentially fatal issues, constitutes a promising option as source of envelope proteins for pseudotyping LV vector particles [154]. Infections by NiV are very rare, making the existence of a pre-existing humoral immunity that would interfere with gene transmission unlikely [109]. Nipahs have two envelope proteins—F and G glycoproteins (NiV-F and Niv-G, respectively). NiV-G is responsible for binding to the viral receptor, while F is the protein that carries out membrane fusion with the same mechanism than MV, as previously described [139] (Figure 3).

The NiV-G glycoprotein consists of a stem domain and a globular head. It forms dimers linked by disulfide bonds which combine in pairs to generate tetramers [155]. Functional pseudotyping of LV vectors by these proteins is possible but it requires the truncation of the cytoplasmic tail of the NiV-F protein. Recently, indeed, a truncated variant mutated in four residues of the cytoplasmic tail (FcΔ22) also shows a ten-fold increased efficiency of pseudotyping [110,156]. Concerning the NiV-G protein, it has been reported that the full-length form can be used, although two truncated forms of the protein, GcΔ33 and GcΔ34, in which have been deleted respectively the 33 and 34 N-ter amino acids, provide optimized pseudotyping [109,156]. It has already been described that shortened cytoplasmic tails of the attachment proteins increase the titers of Paramyxoviridae-pseudotyped LV vectors [157,158]. It is therefore not unexpected that this strategy is transposable to pseudotyping LV vectors by Nipah envelope proteins [109]. The advantages conferred by the truncation can be explained by the location of the truncated forms of the NiV-G proteins in plasma membrane regions far from ephrin-B2, which is normally located at the junction of neighboring cells. For the truncated forms, this limits, at the same time, the cytopathic effect exerted at these junctions and their sequestration by ephrin-B2 molecules expressed in producer cells. This enhances their incorporation into LV vector particles [109].

Attachment of Nipah on the target cell occurs through binding to Ephrin-B2 [159] as a primary receptor, although Ephrin-B3 can also be recognized for attachment albeit with an affinity ten times lower [160]. Ephrin receptors are membrane-associated tyrosine kinase (RTK) receptors with different roles in several biological processes such as neurogenesis and angiogenesis. The main receptor, Ephrin-B2, is highly expressed in the arterial endothelium and, to a lower extent, in pericytes and vascular smooth muscle cells [161,162]. Usually expressed at the cell–cell junctions, Ephrin-B2/Ephrin-B4 interactions are strongly involved in angiogenesis, cell migration and tumor invasion. Pseudotyping LV vectors with wt Nipah envelope proteins would therefore allow to address viral vectors to these areas, potentially interfering with the setting up of the angiogenic processes that are associated with tumor expansion.

Besides these potential applications of LV vectors pseudotyped by the wild-type Nipah envelope proteins, the use of engineered proteins can also be envisaged. Namely, to increase the specificity of the vector pseudotyped by NiV-G, NiV-GcΔ34 has been modified by point mutations in order to abolish binding to Ephrin-B2 [156]. This study showed that combining some mutations can reduce binding to a level below detection, providing a basis for the development of variants with an entirely new tropism. By the insertion of new ligands in the mutated H protein [156], it has been possible to transduce efficiently model cells expressing either CD20 (a marker of B cells), CD8 (a marker of cytotoxic T cells), or EPCAM (a putative marker of early tumor cells [163]) with the same selectivity as reference LV vectors pseudotyped with receptor-targeted measles virus envelope proteins. Thanks to the lower exposure of the population to Nipah virus, engineered NiV envelope proteins could therefore provide an alternative for LV vector pseudotyping with a reduced risk of antibody neutralization of the vector with respect to MV.

### 5.3. Togaviridae: The Pair E1-E2 Dissociates to Trigger Membrane Fusion

#### 5.3.1. Chikungunya Virus

Chikungunya virus (ChikV) is an alphavirus from the *Togaviridae* family. Akin to all togaviruses, its spikes are composed by the E1, E2 and E3 envelope glycoproteins, associated non-covalently. E1 is responsible for pH-dependent viral and cell membrane fusion [164] after the clathrin-dependent endocytosis [165,166] of the virus. E2 carries out the association with cellular receptors [167] while E3 is responsible for the translocation of the spikes to the endoplasmic reticulum. E3 also displays chaperone activity to assist the correct folding of the E2 precursor and, by acting as a clamp, stabilizes the association between E1 and E2 [168,169]. E3 is ultimately cleaved from E2 in the trans-Golgi network. This cleavage is necessary to remove the “clamp” since, upon binding to the target cell at low pH conditions, dissociation of the E2-E1 heterodimer becomes necessary in order to mediate fusion between the viral and host cell membranes during viral entry [170]. Indeed, for entry, once E2 and the target receptor have interacted, dissociation of the E1/E2 complex, probably induced by the acidification of the endosome, is required to trigger fusion [171]. The dissociation exposes a non-polar domain at the E1 apex, which is able to start the fusion by anchoring to the membrane of the endosome. Nevertheless, some studies revealed the existence of alternative entry pathways such as macropinocytosis [172], probably coexisting with the main clathrin-mediated, one (Figure 3).

The tropism conferred by this envelope is not fully defined, although heparan sulfate, integrins and the matrix remodeling-associated protein 8 (Mxra8, a protein expressed on a large panel of cell types) have been shown to be involved in binding of the virus to the cell surface. The widespread expression of these proteins can explain the large variety of symptoms observed during infection with wild-type ChikV: encephalitis and neurological complications [173], arthralgia/myalgia [174] and ophthalmic disorders [175]. The neurological disorders associated with this virus have suggested the use of ChikV-pseudotyped LV vectors for transducing cells of the central nervous system. A study has indeed shown that ChikV-pseudotyped LV vectors efficiently transduce neurons and astrocytes both in vitro and in vivo [176]. Despite these results, it has not been shown that this type of pseudotyping allows specific targeting in vivo. Another receptor, Prohibitin (PHB), has also been described as a target for ChickV [111]. PHB is an evolutionarily conserved and ubiquitously expressed protein. It has a broad range of functions: it has a structural role at the level of the cell membrane, it is involved in transcription, in mitochondrial morphogenesis and apoptosis [177,178]. Even if PHB is overexpressed in some cancers, such as diffuse large B-cell lymphomas [178], also in this case, its widespread expression does not allow it to be used as a target for specific interventions in vivo. Therefore, at least until variants with a more restricted tropism will be developed, the most straightforward therapeutic application for the use of ChickV envelope proteins seems to be the reprogramming of target cells in vitro [176]. Furthermore, the frequent recognition of the ChikV envelope by the immune system, having oriented the research to focus preferentially on another less immunogenic but just as promising as ChikV for genetic manipulations, alphavirus envelope: the one of the Sindbis virus.

#### 5.3.2. Sindbis Virus

The Sindbis virus (SV), similar to ChikV, is an alphavirus from the *Togaviridae* family. Since these viruses are genetically related [179], their envelope proteins have similar structures, with a step of dissociation between E1 and E2 essential for triggering membrane fusion in both cases. Due to the similarity between alphaviruses, the entry mechanism of Sindbis virus is described as being strictly similar to that of ChikV.

Despite their relatedness, the receptors used differ between these two viruses. The most well documented receptor capable of interactions with SV E2 is the 67 kDa non-integrin high affinity laminin receptor, 67LR [115]. 67LR is found in lipid rafts of a wide variety of normal cell types such as intestinal epithelium cells [180], neurons, hematopoietic cells [181] and in some cancer cells as carcinomas [182]. The natural resistance-associated macrophage protein 2 receptor (NRAMP2, also known as DMT1, for divalent metal transporter 1), expressed in almost all cell types [183,184,185], has also been described as being an E2 interactant [116]. As for ChikV, viral entry into the cell follows a clathrin-dependent pathway with the subsequent release of the capsid into the cytoplasm (Figure 3). It is noteworthy that some studies support the idea that Sindbis fusion could occur at the plasma membrane with no need for clathrin-dependent endocytosis [186,187] (Figure 3).

The use of the wt Sindbis virus envelope in pseudotyping for the purposes of viral vectorization in vivo is limited because of its wide tropism. However, the main interest of its envelope proteins comes from their versatility in genetic modifications. Numerous studies have described variants of SV envelope proteins that have been modified by genetic engineering and used to pseudotype LV vectors. In particular, a loop of the E2 protein allows insertion of exogenous motifs without affecting the ability of the SV envelopes to promote viral entry in the target cell. The most successful results obtained so far concern the insertion of the ZZ domain derived from the IgG-binding domain of protein A of *Staphylococcus aureus* between amino acids 71 and 74 of E2 [188]. This strategy made it possible to bind the envelope protein to the Fc fragment of an IgG directed against specific receptors in order to reorient the vector to enter a defined type of cells thanks to the interaction between the ZZ domain and the IgG. Other works aimed at reducing non-specific targeting through abolishing the natural tropism of the virus. The most successful one resulted in the creation of a variant called m168 [189] which was fusion competent but deficient for binding to the natural receptors of SV. The mutations present in the m168 variant are, in addition to the insertion of the ZZ motif in E2, a deletion of the residues 61-64 of E3 and the insertion of four point mutations in E2 (K159A, E160A, E216A and T218A) [190]. Therefore E1, responsible for the fusion of the membranes, was not mutated. The m168 mutations were then combined to coupling an IgG directed against the P glycoprotein that is expressed in the lungs [191]. When the vector was administered by intravenous injection in model mice, the pseudotyped lentiviruses were addressed to the lungs with a low dispersion of the vectors in the rest of the organism. However, this strategy requires a step of non-covalent coupling between the viral particle and the antibodies, which, being a labile interaction, constitutes the critical point of the strategy.

In conclusion, alphaviruses’ wt envelope proteins cannot be used for in vivo approaches, due to their natural broad tropism. However, the marked separation of the binding and of the fusion functions, carried out by E2 and by E1, respectively, allows envisaging modifications to the E2 protein so as to allow the most specific interactions possible with the desired targets without affecting the fusion step. The main concern is the preservation of the association between E1 and E2 at the surface of the viral particle.

## 6. Concluding Remarks

Viral vectorization for gene therapy is a cutting-edge technique whose development has not yet shown its full potential. Even if several virus-based vectors have been identified as being potentially useful for these approaches, so far, the choice of preference regularly falls on a handful of vectors, among which are lentiviral vectors. A feature of LV vectors that makes them particularly promising is their possibility to uptake envelope proteins from other viruses. These exogenous envelopes can either possess a limited natural tropism that can match the tropism required for the intervention or can have a widespread tropism. In this latter case, they cannot be employed the way they are but, if they have a high plasticity with respect to genetic engineering, they can be used as starting points for the elaboration of artificial envelopes with a specific tropism of interest for the approach followed. LV vectors play the role of mannequins for these approaches for both the “prêt à porter” envelopes and for the “tailor-made” ones, being extremely useful for the development of these therapeutic approaches. Furthermore, emerging viruses may provide interesting alternatives to the envelopes already studied. In this sense, viruses responsible for today’s illnesses may become part of tomorrow’s therapies.

## Figures and Tables

**Table 1:** Overview table summarizing the main characteristics of the pseudotypes discussed in this review.

Original Virus	Pseudotype	Main Characteristics of the Pseudotyped LV	Natural Cell Tropism	Receptor	Transduction Efficiency	References
Vesicular Stomatitis virus	VSV-G	Quasi-universal tropism, high efficiency	Broad	LDL-R	High	[95,96]
Rabies virus	RabV-G	Natural ability to efficiently targets neurons	Neurons	nAChR, CD56, p75NTR, mGluR2	Up to 50%	[97,98,99,100]
Measle virus	H/F	High efficiency, tolerant to peptide insertion, can be neutralized by vaccines	B cells, T cells, Epithelial cells, Dendritic cells, HSPC	CD46, SLAM, nectin-4	Up to 50–70%	[101,102,103,104,105,106,107,108]
Nipah virus	G/F	Low prevalence: low neutralization hazard	Pericytes, tumor endothelium	EphrinB2, EphrinB4	20–40%	[109,110]
Chickungunya virus	E1/E2	Versatile basis for engineering/reprograming	Broad	PHB1, Mxra8, integrins, Heparan sulfates	Low on non-adherent cells, high on adherent cells (related to VSV-G)	[111,112,113,114]
Sindbis virus	E1/E2	Versatile basis for engineering/reprograming, Low immunogenicity	Broad	67LR, NRAMP2	Variable	[115,116]
